# Flourishing, social participation and reintegration difficulties: a 2-year cohort study of US military veterans with invisible injuries

**DOI:** 10.3389/fresc.2026.1720234

**Published:** 2026-06-10

**Authors:** Nicholas A. Rattray, Mindy Flanagan, Richard M. Frankel, Marina Kukla

**Affiliations:** 1 Department of Internal Medicine and Geriatrics, Indiana University School of Medicine, Indianapolis, IN, United States; 2 VA HSR Center for Health Information and Communication, Roudebush VAMC, Indianapolis, IN, United States; 3 Center for Health Services Research, Regenstrief Institute, Indianapolis, IN, United States; 4 Department of Psychology, Indiana University-Indianapolis, Indianapolis, IN, United States

**Keywords:** longitudinal, mental health, observational, post-traumatic stress, quality of life, rehabilitation, reintegration, veterans

## Abstract

**Introduction:**

Longitudinal research examining community reintegration among military veterans with invisible injuries remains limited, despite widespread recognition that post-separation adjustment encompasses multiple interconnected domains including mental health, physical health, social functioning, and overall well-being. This study investigated associations between changes in reintegration outcomes and changes in health-related quality of life, social support, and flourishing over a two-year period among U.S. military veterans with invisible injuries who separated from service within the previous five years.

**Methods:**

Seventy-five veterans diagnosed with invisible injuries (post-traumatic stress disorder, anxiety, depression, traumatic brain injury, or adjustment disorder) completed assessments at baseline, 6, 12, 18, and 24 months. Reintegration was measured using the Military to Civilian Questionnaire (M2C-Q) and Community Reintegration of Injured Service Members computer-adaptive test (CRIS-CAT). Health and well-being outcomes included the Veterans RAND Health Survey (VR-12), Patient Health Questionnaire-15 (PHQ-15), Multidimensional Scale of Perceived Social Support (MSPSS), and Secure Flourishing Index (SFI). Mixed-effects models examined associations between changes in reintegration measures and psychosocial outcomes while controlling for demographic characteristics and PTSD symptoms.

**Results:**

M2C-Q scores remained stable across waves (F(4,236) = 1.73, *p* = .14), while CRIS-CAT Participation scores improved significantly (F(4,238) = 10.10, *p* < .001). Improvements in community participation (CRIS-CAT) were positively associated with subsequent secure flourishing and physical health quality of life. Reductions in reintegration difficulties (M2C-Q) positively influenced social support, secure flourishing, somatic symptoms, and mental health quality of life. Social support and secure flourishing increased significantly between 6 and 24 months, while physical and psychological symptoms remained relatively stable throughout the study period.

**Discussion:**

Findings demonstrate that improvements in community participation and reductions in reintegration difficulties precede positive changes in psychosocial outcomes and physical symptoms. The relative stability of reintegration challenges alongside improvements in community participation suggests these constructs may represent distinct aspects of veteran adjustment. Results suggest the potential for interventions targeting community engagement and social connection during the critical transition period, emphasizing holistic approaches that address both functional participation and subjective reintegration experiences to enhance long-term veteran well-being.

## 1 Introduction

To better understand potential factors associated with challenges military veterans face with community reintegration, prospective research with repeated measurements that can track changes over time is beneficial. While there has been some research focused on early readjustment issues, many studies are cross-sectional in nature and typically focused on a limited range of psychosocial outcomes. Among those who report difficulties with adjusting to civilian life, common issues include decreased mental and physical health, financial strain, employment challenges, and lower functioning in social relationships (1). Reviews have demonstrated that many studies focus on readjusting to life after deployment, but less research has focused on the relationship with time following permanent separation from military service. The extant literature that focuses on post-military life have found the issue of loss, including missing support from military peers, identity loss, and purpose (2). Relative consensus can be found in reviews of key domains that shape reintegration: mental health, identity, social functioning, physical health, finances, and healthcare access (3, 4). Evidence has emerged identifying stability with employment or financial issues as well as well-functioning social relationships as critical for successful reintegration (5, 6). Numerous researchers have called for longitudinal studies, moving beyond a focus on mental health alone and the inclusion of issues related to financial issues, parenting, and quality of life (2, 7, 8).

The longer-term consequences of military service on neuropsychological and social outcomes have been a source of debate, with questions that remain about the persistence of adverse symptoms related to PTSD and traumatic brain injury. In studies involving PTSD, some previous research points to lessening of symptoms with time (9, 10). However, Eekhout and colleagues have shown that researchers reported deployment effects in the short term (i.e., within 1 year after deployment), and some did so for the long-term effect of deployment on development of post-traumatic stress symptoms (i.e., 2 or 3 years after deployment) but none looked at multiple timepoints or follow up beyond 3 years (11). Multiple researchers have identified development trajectories, including “resilient,” “recovery,” and “delayed onset” trajectories, with some studies also finding a chronic trajectory (12, 13). Another epidemiological study found that that adverse stress reactions cannot necessarily be expected to dissipate over time and may increase (14).

The Veteran Metrics Initiative takes a longitudinal approach to military-to-civilian issues by evaluating key domains (vocational, financial, social, health) based on a nationwide cohort of US veterans (15). In response to limitations of existing studies, this initiative focuses on change over time, adopts a comprehensive approach to assessing well-being and program utilization, and works in partnership across institutions and funders. Reports from initial waves of data collection have addressed veterans' preferences for educational and employment resources services (16), reporting on gender differences in moral injuries (17) and psychological health, trajectories of suicidal ideation (18), and broad concerns and well-being in the first year after separation (19). Earlier longitudinal studies on post-9/11 veterans found high quality of life and family functioning among veterans with PTSD, but unique gender differences in comorbid mental health symptoms (20).

While many studies argue persuasively for early intervention, questions remain about how the timing of interventions or preventive strategies coincide with patient needs and wider patterns of mental health and functional challenges related to readjustment and civilian reintegration. Most studies focus on specific diagnoses such as PTSD or TBI but few approach reintegration difficulties through repeated measure designs. A recent evidence synthesis conducted by the VHA concluded that few studies track longitudinal outcomes and that there is a dearth of studies examining the effectiveness of existing interventions that directly address readjustment issues (21).

Given these gaps in longitudinal research on post-military trajectories, it is essential to clarify three core constructs underpinning Veteran reintegration and well-being. Flourishing is widely examined within positive psychology, with models such as Seligman's PERMA framework highlighting dimensions of positive emotion, engagement, relationships, meaning, and accomplishment (22). However, the present study uses VanderWeele's multidomain Flourishing Index, which conceptualizes well-being across six domains—life satisfaction, mental and physical health, meaning and purpose, character and virtue, close social relationships, and financial/material stability—and aligns directly with our measurement approach (23, 24). VanderWeele and Lomas note that the well-being literature is often conceptually fragmented, prompting the need for integrated, multidomain frameworks. such as the Flourishing Index, that provide clearer boundaries and greater coherence across studies (25). Social participation aligns with the World Health Organization's International Classification of Functioning, Disability and Health, which defines participation as involvement in life situations; recent work suggests specifying participation through social roles to capture societal involvement more precisely (26, 27). In terms of reintegration, Among the various reintegration challenges described in prior research, Sayer et al. provide the most widely used operational definition through the Military to Civilian Questionnaire (M2C-Q), which characterizes reintegration difficulties across multiple spheres of daily life. Grounding the study in these interrelated constructs provides a coherent framework for examining two-year changes in reintegration, well-being, and participation in the early post-separation period.

Our baseline cross-sectional analyses applied this framework shortly after separation and observed associations between reintegration difficulty and social support, mental health, and flourishing (28). The present study extends the framework longitudinally over two years beginning 0–5 years after separation from military service. We aimed to understand associations between changes in reintegration outcomes and changes in outcomes related to mental health, physical health, social support, life satisfaction and community reintegration. Our prior cross-sectional assessment found that fewer difficulties with reintegration were associated with greater social support, higher mental health-related quality of life, and lower levels of post-traumatic stress and somatic symptoms. We aim in the present study to understand associations in these outcomes from a longitudinal perspective. We postulated that Veterans whose reintegration improved would be associated with increased social support, better overall physical and mental health functioning, and improved flourishing.

## 2 Materials & methods

### 2.1 Participants

The study sample consisted of US Veterans enrolled prospectively in a project designed to examine community reintegration outcomes. Methods of recruitment and assessment have been described (28). Veterans within five years of separating from military service were identified using the VA administrative databases. Eligible participants (a) completed military service after 2001, (b) separated from service within the last 60 months, and (c) have a diagnosed invisible injury, including post-traumatic stress disorder, anxiety, depression, traumatic brain injury, adjustment disorder. Invisible injuries were defined using ICD-10 and ICD-9 diagnostic codes recorded in the VA EHR, encompassing PTSD and trauma-related disorders (F43.10, F43.12, F43.20–F43.25; ICD-9: 309.xx), major depressive and persistent depressive disorders (F32.x, F33.x; ICD-9: 296.2x/296.3x), anxiety disorders (F41.x; ICD-9: 300.00), and adjustment disorders (F43.2x; ICD-9: 309.xx). Additional qualifying conditions included TBI and associated neurocognitive symptoms (S06.0X0A/S06.0X1A; F06.3x; F07.9). The study was approved by the Indiana University institutional review board and VA research and development committee.

### 2.2 Recruitment

Based on data in the VA electronic health record, the study team extracted a list of eligible patients. A total of 75 participants completed a baseline assessment. After obtaining consent, trained researchers collected demographic information and administered a set of measures described below. Participants received a $25 gift card for completing the assessment. Follow up interviews were completed at 6 months (*n* = 63), 12 months (*n* = 61), 18 months (*n* = 61), and 24 months (*n* = 55). Baseline recruitment started in January 2019 and was completed in February 2021. The last 24-month interviews were completed in January 2023.

### 2.3 Measures

#### 2.3.1 Veteran reintegration

We employed two distinct instruments to evaluate Veteran reintegration. The Military to Civilian questionnaire (M2C-Q), is a concise self-report scale designed to gauge reintegration difficulties, as validated by Sayer et al. (29). The M2C-Q explores dimensions such as social relations, productivity in education, work, domestic life, community engagement, perceived meaning in life, self-care, and leisure. Respondents rated 16 items on a 5-point scale (0–4), with total scores ranging from 0 to 4 and higher scores indicating greater reintegration challenges. The M2C-Q was developed with sample of U.S. military Veterans to assess reintegration difficulties within the past 30 days, distinguishing it from measures targeting psychological transition and cultural adjustment after permanent separation from military service (30, 31). In a survey of 1,291 Veterans from the post-9/11 period, the mean M2C-Q score was 1.41 (1).

The Community Reintegration Scale (CRIS), was developed by VA rehabilitation researchers to measure community reintegration and participation within the framework of the International Classification of Functioning, Disability, and Health (ICF) (5, 32). We used the computer-adapted version, known as the CRIS-CAT, which has demonstrated robust validity (33). The Participation subscale assesses the extent and frequency of participation. Items are rated on a 7-point scale (1 = More than once per day, 7 = Never) with higher scores on the CRIS-CAT subscales signify greater levels of community reintegration (range = 0–100). The item pool for the Participation subscale includes 77 items; respondents are asked a minimum of 10 items and maximum of 20 items. Field testing of the CRIS-CAT with 332 OEF/OIF Veterans resulted in mean score of 48.7 (33).

#### 2.3.2 Health and well-being

To assess health and well-being outcomes, we employed several reliable measures. The Secure Flourishing measure encompasses six domains associated with sustained well-being (23), validated in a cross-cultural study (34). These domains include happiness, mental and physical health, meaning and purpose, character, social relationships, and financial stability. The Secure Flourishing Index (SFI) is computed as the average of 12 items, with each item assessed on an 11-point scale (0–10). Higher scores indicate greater flourishing (35–37).

The Veterans RAND Health Survey (VR-12) is a self-administered instrument that measures health-related quality of life (38). The questionnaire consists of 12 items that assess eight physical and mental health domains, which can be summarized into two separate scores: the physical component summary (PCS) and the mental health summary (MCS). Scores for each domain range from 0 to 100 with higher scores indicating better health (39). Notably, both the PCS and MCS reliably detect incremental effects of disease burden on health-related quality of life (i.e., hypertension, diabetes, angina, depression) (40).

The Patient Health Questionnaire-15 (PHQ-15) encompasses 15 items designed to evaluate the severity of somatic symptoms, including pain, sleep disturbances, headaches, fatigue, and other common clinical manifestations (41). Since somatic symptom burden significantly influences overall impairment perception, it is closely related to health-related quality of life. PHQ-15 items are assessed on a 3-point scale (0 = not at all, 1 = a little, 2 = a lot) and the scale is scored by summing all responses (range=0–30). Scores exceeding 15 have been associated with increased healthcare utilization and greater impairment in military samples (42).

The Multidimensional Scale of Perceived Social Support (MSPSS) is a self-reported measure featuring 12 items that assess perceptions of support from three different sources: significant others, family, and friends (43, 44). Respondents rate items on a 7-point scale (1 = Strongly disagree, 7 = Strongly agree). The MSPSS has been widely used across clinical and non-clinical samples, demonstrating robust reliability and validity across various age groups. Scores ranging from 1 to 2.9 denote low support, 3 to 5 indicate moderate support, and scores from 5.1 to 7 signify high support.

### 2.4 Analyses

Descriptive statistics were calculated for all measures. Change in reintegration score was calculated as the difference from baseline score at each wave. Participants missing a wave of data were compared to participants with no missing waves of data on baseline reintegration scores using independent t-tests (for both reintegration measures) with a correction for inequality of variances, when appropriate.

Random effects models, also known as mixed-effects models, address the correlated nature of observations on the same individuals at multiple time points. In a longitudinal context, the model describes variation in observations within individuals over time and between-individual characteristics. Fixed effects represent the average relationship between predictor variables and outcome variables. In the present set of analyses, predictors included covariates (gender, months since military separation, PCL score), wave, baseline reintegration, and change from baseline reintegration. Wave, or time point, was included to account for average change in outcome variables during the 24-month study period attributable to secular trends. The inclusion of both baseline reintegration and change from baseline reintegration captured cross-sectional differences at study initiation from within-participant changes that were associated with outcomes. Outcome variables were social support, PHQ-15, MCS, PCS, and secure flourishing.

A model was estimated for each outcome and each reintegration measure, with individual-level intercepts as random effects. Initially, a series of models was estimated to determine the set of covariates to include. All covariates reaching statistical significance (*p* < .05) were retained in subsequent models. Though time since military separation was not significant, it was included in models as it was related to the sampling procedure. For the primary set of analyses, random effects models relating change in reintegration to the five outcome variables were estimated using maximum likelihood and compound symmetric covariance structure. Additionally, similar models were estimated that tested a time trend as predicting reintegration. *P*-values were adjusted for multiple comparisons using the Holm-Bonferroni correction (45).

## 3 Results

**Figure 1:**
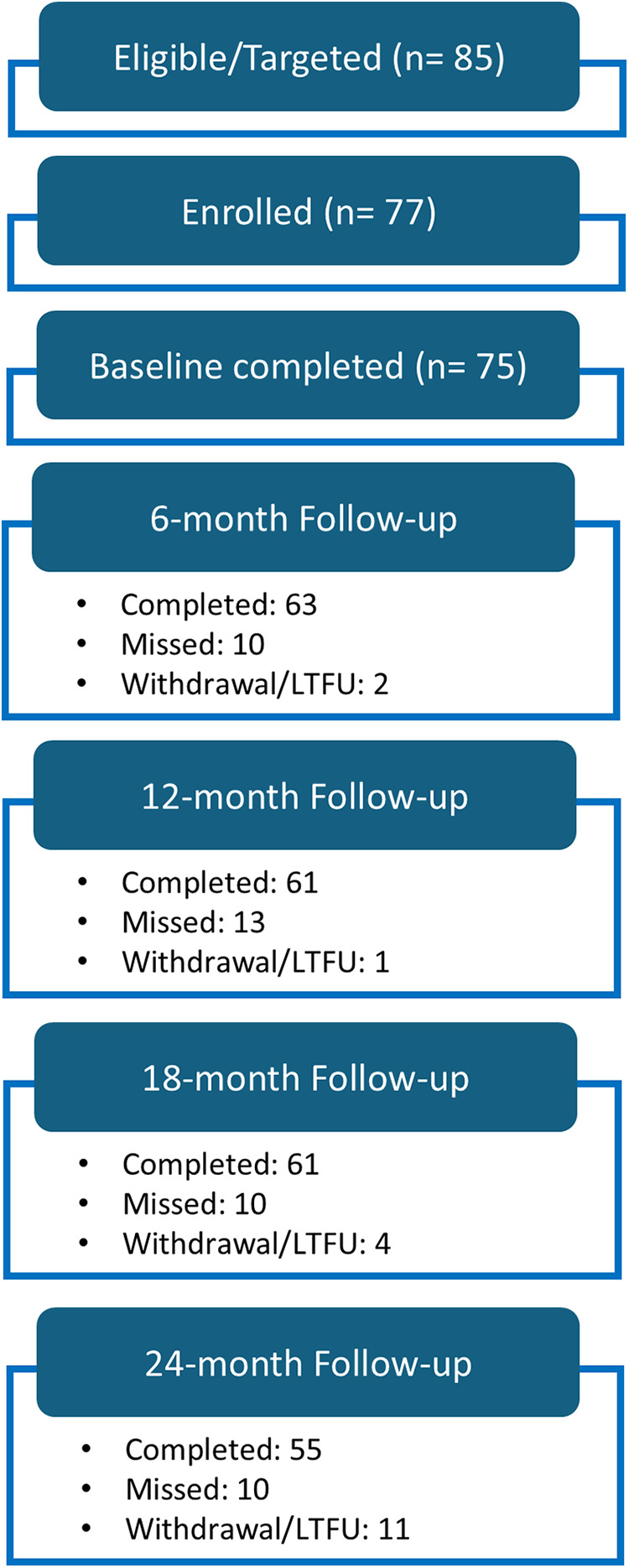
Participant flow through study waves. Participant flow diagram detailing recruitment, enrollment, completion and longitudinal follow-up across 24 months. The figure shows data completion, missing data due to missed appointments, and cumulative attrition.

A total of 75 Veterans participated at baseline. Of these, 61% were less than 40 years old, 79% were male, 85% were White, 9% were Hispanic (see Table 1). A full description of sample characteristics has been reported elsewhere (28). At baseline, Veterans had an average of 41.64 (SD = 7.95) on the CRIS-CAT Participation Scale and 1.49 (SD = 0.73) on the M2C-Q. (See Table 2 for mean reintegration scores at each wave). Participants that missed at least one wave of data collection (36%, 27/75) did not significantly differ from those with complete data on baseline M2C-Q (t(7) = 1.84, *p* = .07) or CRIS-CAT Participation scores (t(38) = 0.79, *p* = .44). Retention is the study is described in Figure 1. Participants with missing data also did not differ in gender, age, race, ethnicity, PTSD symptoms, and months since military separation from those without missing data. A total of 64% (48/75) participants completed all five waves of data collection, 11% (8/75) completed 4 waves, 11% (8/75) completed 3 waves, 11% (8/75) completed 2 waves, and 4% (3/75) completed only one wave. M2C-Q scores across waves were relatively stable and did not significantly change over time (F(4, 236) = 1.73, *p* = .14); in contrast, CRIS-CAT Participation scores were significantly related to wave (F(4, 238) = 10.10, *p* < .001), with improving scores across waves (see Table 1).

**Table 1:** Sample characteristics by wave.

Characteristic	Wave				
	Baseline(*N* = 75)	6 months (*N* = 63)	12 months (*N* = 61)	18 months (*N* = 61)	24 months (*N* = 55)
Age group					
<40	46 (61%)	37 (59%)	37 (61%)	36 (59%)	32 (58%)
40–54	28 (37%)	25 (40%)	23 (38%)	24 (39%)	22 (40%)
55+	1 (1%)	1 (2%)	1 (2%)	1 (2%)	1 (2%)
Gender					
Female	16 (21%)	14 (22%)	14 (23%)	14 (23%)	13 (24%)
Male	59 (79%)	49 (78%)	47 (77%)	47 (77%)	42 (76%)
Race					
American Indian	1 (1%)	1 (2%)	1 (2%)	1 (2%)	1 (2%)
Asian	0 (0%)	0 (0%)	0 (0%)	0 (0%)	0 (0%)
Black	10 (13%)	8 (13%)	9 (15%)	8 (13%)	9 (16%)
Pacific Islander	0 (0%)	0 (0%)	0 (0%)	0 (0%)	0 (0%)
White	64 (85%)	54 (86%)	51 (84%)	52 (85%)	45 (82%)
Multiple Races	0 (0%)	0 (0%)	0 (0%)	0 (0%)	00 (0%)
Hispanic					
No	68 (91%)	57 (90%)	55 (90%)	55 (90%)	51 (93%)
Yes	7 (9%)	6 (10%)	6 (10%)	06 (10%)	4 (7%)
Separation group					
0–12 months	20 (27%)	18 (29%)	16 (027%)	15 (25%)	15 (028%)
13–35 months	25 (34%)	22 (35%)	22 (37%)	22 (37%)	20 37%)
36–60 months	29 (39%)	22 (35%)	22 (37%)	23 (38%)	19 (35%)
Household financial situation					
Comfortable	46 (61%)	48 (76%)	45 74%)	49 (80%)	41 (75%)
Just enough to make ends meet	22 (29%)	11 (17%)	14 (23%)	9 15%)	10 (18%)
Not enough to make ends meet	6 (8%)	4 (6%)	2 (3%)	3 (5%)	4 (7%)
Prefer to not say	1 (1%)	0 (0%)	0 (0%)	0 0%)	0 (0%)
Marital status					
Divorced	12 (16%)	10 (16%)	10 (16%)	10 (16%)	9 (16%)
Living together	2 (3%)	2 (3%)	2 (3%)	2 (3%)	2 (4%)
Married/civil union/domestic partnership	40 (53%)	35 (56%)	33 (54%)	34 (56%)	32 (58%)
Never married	16 (21%)	12 (19%)	14 (23%)	11 (18%)	10 (18%)
Separated	5 (7%)	4 (6%)	2 (3%)	4 (7%)	2 (4%)

**Table 2:** Mean (SD) for reintegration measures by wave.

Reintegration Measure	Wave				
	Baseline (*N* = 75)	6 months (*N* = 63)	12 months (*N* = 61)	18 months (*N* = 61)	24 months (*N* = 55)
M2C-Q	1.49 (0.73)	1.37 (0.79)	1.29 (0.72)	1.34 (0.79)	1.22 (0.71)
CRIS-CAT Participation	41.64 (7.95)	43.04 (7.53)	44.35 (7.38)	44.67 (7.38)	45.91 (7.15)
CRIS-CAT Perceived Limitation	43.71 (4.15)	44.58 (5.17)	45.90 (6.26)	46.65 (6.68)	47.96 (6.27)
CRIS-CAT Satisfaction	42.93 (4.32)	43.97 (5.45)	46.15 (7.87)	45.18 (5.95)	45.74 (7.12)

As shown in Tables 3, 4, baseline reintegration scores were related to nearly all outcome variables, such that higher reintegration was associated with better psychosocial outcomes. Changes in reintegration from baseline were related to psychosocial outcomes as well, though not for all outcomes. Change in the CRIS-CAT Participation score from baseline at each time point was positively related to secure flourishing; and, change in M2C-Q score from baseline was negatively related to social support and secure flourishing. Conversely, change in M2C-Q from baseline was positively related to PHQ-15 score suggesting that more reintegration difficulties were associated with more physical symptoms. Change in the CRIS-CAT Participation score was significantly and positively related to VR-12 PCS score; however, change in M2C-Q score was not related to VR-12 PCS score. In contrast, change in the M2C-Q score was significantly and inversely related to VR-12 MCS but change in CRIS-CAT Participation score was not related to VR-12 MCS.

**Table 3:** Summary results from random effects models predicting individual and interpersonal outcomes from change in M2C-Q score (*n* = 72).

Variable	PHQ-15		PCS		MCS		Secure flourishing		MSPSS	
	Parm Est	Std Err	Parm Est	Std Err	Parm Est	Std Err	Parm Est	Std Err	Parm Est	Std Err
Intercept	2.93	1.79	60.99***	5.00	54.34***	3.56	9.06***	0.47	6.37***	0.43
Age	0.05	0.04	−0.29	0.11	0.16	0.08	0.01	0.01	0.01	0.01
Gender (Female)	0.64	0.87	6.06	2.42	−5.91**	1.70	−0.02	0.23	−0.08	0.21
Months since separation	0.03	0.02	−0.02	0.05	−0.02	0.04	0.00	0.01	−0.01	0.01
PCL-5	0.06**	0.02	−0.02	0.05	−0.24***	0.05	−0.02***	0.01	−0.00	0.01
Wave 2 vs. 5	−0.80	0.43	−0.68	1.20	−1.25	1.20	−0.42***	0.11	−0.30*	0.11
Wave 3 vs. 5	−0.25	0.43	−1.99	1.20	0.55	1.20	−0.23	0.11	0.07	0.11
Wave 4 vs. 5	−0.67	0.43	0.04	1.19	−0.75	1.19	−0.10	0.11	−0.08	0.11
Baseline M2C-Q	2.61***	0.61	−3.14	1.71	−5.99***	1.33	−1.10***	0.16	−0.65***	0.16
M2C-Q Change	1.43**	0.44	−0.59	1.22	−4.22***	1.11	−0.53***	0.11	−0.38**	0.11

*p < .05, **p < .01, ***p < .001; MPSS, Multidimensional Scale of Social Support; MCS, Mental Health Component (VR-12); PCS, Physical Health Component (VR-12); PHQ-15, Patient Health Questionnaire-15; PCL-5, Posttraumatic Stress Disorder Checklist for the DSM-5.

**Table 4:** Summary results from random effects models predicting individual and interpersonal outcomes from change in CRIS-CAT participation score (*n* = 72).

Variable	PHQ-15		PCS		MCS		Secure flourishing		MSPSS	
	Parm Est	Std Err	Parm Est	Std Err	Parm Est	Std Err	Parm Est	Std Err	Parm Est	Std Err
Intercept	14.61***	3.04	27.51**	8.07	31.63***	6.92	4.30***	0.87	4.11***	0.82
Age	0.03	0.04	−0.26	0.11	0.20	0.09	0.02	0.01	0.02	0.01
Gender (Female)	0.63	0.86	7.26**	2.30	−6.10**	1.86	−0.03	0.26	−0.10	0.23
Months since separation	0.02	0.02	0.00	0.05	0.00	0.04	0.00	0.01	−0.01	0.01
PCL-5	0.08***	0.02	0.05	0.04	−0.32***	0.04	−0.03***	0.00	−0.01*	0.00
Wave 2 vs. 5	−0.91	0.45	0.30	1.19	−1.16	1.23	−0.38**	0.11	−0.27	0.11
Wave 3 vs. 5	−0.39	0.45	−1.47	1.16	0.79	1.21	−0.17	0.11	0.11	0.11
Wave 4 vs. 5	−0.67	0.44	0.08	1.15	−0.78	1.19	−0.10	0.11	−0.07	0.11
Baseline Participation score	−0.18**	0.05	0.56***	0.14	0.34*	0.12	0.07***	0.02	0.03*	0.01
Participation score change	−0.09	0.04	0.49***	0.11	0.16	0.11	0.03*	0.01	0.03	0.01

*p < .05, **p < .01, ***p < .001; MPSS, Multidimensional Scale of Social Support; MCS, Mental Health Component (VR-12); PCS, Physical Health Component (VR-12); PHQ-15, Patient Health Questionnaire-15; PCL-5, Posttraumatic Stress Disorder Checklist for the DSM-5.

Significant increases over time in social support and secure flourishing were observed as 24 months scores for each were significantly higher than corresponding scores at 6 months. However, change in PHQ-15, VR-12 MCS, and VR-12 PCS did not have a significant effect by observation timepoint, suggesting that physical and psychological symptoms remained relatively stable, on average, during the study period.

## 4 Discussion

To better understand factors that affect how Veterans readjust to civilian life following military service, this study examined changes in reintegration outcomes and various factors, including mental and physical health, social support, life satisfaction, and community reintegration. The study sample consisted of U.S. Veterans who had separated from the military within the last five years and had been diagnosed with invisible injuries, such as post-traumatic stress disorder, anxiety, depression, or traumatic brain injury. Compared to most Veteran studies, this was a younger sample, with 61% under the age of 40. While M2C-Q scores remained stable across waves, CRIS-CAT participation scores significantly improved over time. The relative stability of the M2C-Q during this 24-month period suggests that challenges and adjustments related to reintegration may remain constant for many Veterans. Persistent barriers to reintegration may be due to the pervasive nature of invisible injuries on reintegration as assessed by the M2C-Q (19, 46). For example, as included in the M2C-Q, productivity in education, work, and domestic life, perceived meaning in life, and self-care could require considerable time to experience successes. However, the slight increase in the CRIS-CAT Participation Score suggests that perceived reintegration challenges could co-exist with improvements in community participation and engagement. It is also possible that increases in CRIS-CAT are related to decreased restrictions from the COVID-19 pandemic. One interpretation is that improvements in participation precede global improvements that are better detected by the M2C-Q.

The relationship between changes in reintegration scales and psychosocial outcomes is especially significant. The positive association of improved CRIS-CAT Participation score with social support suggests that community participation contributes to better psychosocial outcomes. Similarly, improvements in reintegration difficulties as assessed by the M2C-Q were positively associated with social support and secure flourishing. These findings are consistent with our previous cross-sectional research that revealed an association between reintegration with both social support and individual flourishing (28). Likewise, the association between changes in reintegration and physical symptoms (as measured by the PHQ-15) suggested that improvements in reintegration may have corresponding improvements in physical symptoms.

Results demonstrate that levels of flourishing improved across the study period. Compared to other approaches to measure well-being, flourishing emphasizes constructs that include material context: that is, where “all aspects of a person's life are good including the contexts in which that person lives” (25). In addition to financial issue, the results reinforce the importance of social support, which has been shown to be associated with high well-being (46) and may function in a protective fashion (47, 48). The use of two measures with slightly different ways of conceptualizing reintegration allowed for more nuanced analyses, which was apparent when comparing mental and physical quality of life. Improvements in the community participation (CRIS-CAT) was found to be positively associated with physical functioning/QoL. Further, decreased reintegration difficulties were associated with increased mental functioning/QoL. Efforts at screening Veterans for issues related to readjustment may choose to include both physical and mental health items, as well as social support.

These findings build on and extend our prior program of research examining reintegration, participation, and flourishing among Veterans with invisible injuries. In our earlier mixed methods work, we identified how reintegration challenges shape subjective well-being, social functioning, and life satisfaction during the transition to civilian life, emphasizing the need for participation-focused supports (49). Subsequent cross-sectional analyses demonstrated strong associations between reintegration, perceived health, social support, and flourishing (28), while more recent work has highlighted the importance of community participation and meaning-making processes in sustaining well-being over time (50). Additional analyses using longitudinal qualitative and survey data have underscored how sustained social connection and participation trajectories influence recovery and flourishing during post-military readjustment (51) as well as how moral injury affects transitions (52). The present study reinforces and extends these findings by showing that within-person improvements in community participation and reductions in reintegration difficulties correspond with gains in flourishing and social support. Taken together, these converging lines of evidence point to the importance of rehabilitation and transition programs that integrate supports for social participation, identity reconstruction, and contextual well-being as core elements of reintegration care.

Several strengths and limitations are worth considering. The sample size limits the generalizability of the findings to a broader population of US Veterans and may have limited statistical power to detect small effects. The study was observational with no comparison group which limits claims about causal inference. However, post-9/11 Veterans who were up to five years post separation were recruited, offering a broader window to observe changes in reintegration and other self-reported outcomes. The sample reflects Veterans engaged in care within specific VA settings. Reintegration experiences and social participation may vary across VA facilities, geographic regions, and community contexts, including differences in local resources and social determinants of health that were not fully captured. Retention in the study was strong given that data collection occurred in 2020–2022 during the COVID-19 pandemic: it is unknown whether participation scores or other outcomes related to social interactions would have been different in the absence of COVID-19 restrictions and subsequent reopening conditions.

This study provides valuable insights into the challenges Veterans face during their transition to civilian life, emphasizing the importance of psychosocial factors, community reintegration, and well-being in this process. These findings can inform the development of interventions and support programs tailored to the specific needs of Veterans during the crucial period of reintegration into civilian society.

## Data Availability

The datasets generated and/or analyzed during the current study are not publicly available due to Department of Veterans Affairs (VA) data security and privacy regulations, but may be made available from the corresponding author upon reasonable request and with appropriate approvals, including a VA data use agreement and institutional review board authorization, where applicable.
